# Comparative Genomics of *Mycoplasma synoviae* and New Targets for Molecular Diagnostics

**DOI:** 10.3389/fvets.2021.640067

**Published:** 2021-02-19

**Authors:** Bin Xu, Xi Chen, Fengying Lu, Yu Sun, Huawei Sun, Jingfeng Zhang, Liya Shen, Qunxing Pan, Chuanmin Liu, Xiaofei Zhang

**Affiliations:** ^1^Key Laboratory of Veterinary Biological Engineering and Technology of Ministry of Agriculture, Institute of Veterinary Medicine, Jiangsu Academy of Agricultural Sciences, Nanjing, China; ^2^National Center for Engineering Research of Veterinary Bio-products, Jiangsu Academy of Agricultural Sciences, Nanjing, China; ^3^Ministry of Education (MOE) Joint International Research Laboratory of Animal Health and Food Safety, College of Veterinary Medicine, Nanjing Agricultural University, Nanjing, China

**Keywords:** *Mycoplasma synoviae*, comparative genomics, core-pan genes, qPCR, molecular diagnostics

## Abstract

*Mycoplasma synoviae* is an important pathogen of poultry, causing significant economic losses in this industry. Analysis of the unique genes and shared genes among different *M. synoviae* strains and among related species is helpful for studying the molecular pathogenesis of *M. synoviae* and provides valuable molecular diagnostic targets to facilitate the identification of *M. synoviae* species. We selected a total of 46 strains, including six *M. synoviae* strains, from 25 major animal (including avian) *Mycoplasma* species/subspecies that had complete genome sequences and annotation information published in GenBank, and used them for comparative genomic analysis. After analysis, 16 common genes were found in the 46 strains. Thirteen single-copy core genes and the 16s rRNA genes were used for genetic evolutionary analysis. *M. synoviae* was found to have a distant evolutionary relationship not only with other arthritis-causing mycoplasmas, but also with another major avian pathogen, *Mycoplasma gallisepticum*, that shares the major virulence factor *vlhA* with *M. synoviae*. Subsequently, six unique coding genes were identified as shared among these *M. synoviae* strains that are absent in other species with published genome sequences. Two of the genes were found to be located in the genetically stable regions of the genomes of *M. synoviae* and were determined to be present in all *M. synoviae* isolated strains (*n* = 20) and *M. synoviae*-positive clinical samples (*n* = 48) preserved in our laboratory. These two genes were used as molecular diagnostic targets for which SYBR green quantitative PCR detection methods were designed. The two quantitative PCR methods exhibited good reproducibility and high specificity when tested on positive plasmid controls and genomic DNA extracted from different *M. synoviae* strains, other major avian pathogenic bacteria/mycoplasmas, and low pathogenic Mycoplasma species. The detection limit for the two genes was 10 copies or less per reaction. The clinical sensitivity and specificity of the quantitative PCR methods were both 100% based on testing chicken hock joint samples with positive or negative *M. synoviae* infection. This research provides a foundation for the study of species-specific differences and molecular diagnosis of *M. synoviae*.

## Introduction

*Mycoplasma synoviae* is an important poultry pathogen. Although it does not directly lead to death in poultry, it causes infectious synovitis, respiratory disease, growth retardation, decrease in egg production, and the production of deformed eggs, leading serious economic losses in the poultry industry (1–3). Thus, far, the best way to prevent diseases caused by *M. synoviae* is pathogen eradication; however, this is very expensive and may be cost-prohibitive for farms in many countries and regions. Serological and molecular diagnosis, control using antibiotics, and immunization are generally implemented in farms (4–6).

At present, two commercial attenuated live vaccines are available to protect against *M. synoviae*. One of the most widely used in the world is the temperature-sensitive MS-H strain, which was developed by the chemical mutagenesis of an Australian field strain 86079/7NS (7). Another vaccine is the NAD-independent MS1 vaccine strain, which was obtained spontaneously during *in vitro* passages of the type strain WVU 1853 (ATCC 25204, NCTC 10124) (6). The whole genome sequence and annotation information of MS-H has been published in GenBank under accession no. NZ_CP021129, while the relevant information of MS1 is not yet available. Comparative genome analysis of the vaccine strain and its wild-type parent strain can be used to find possible pathogenesis-related genes. For example, the *obg* gene, which may affect the temperature sensitivity of the MS-H strain, as well as the possible virulence-related gene *oppF*, were found via comparative genome analysis of the vaccine strain MS-H and its parent strain 86079/7NS (8–10). However, a comparative genomic analysis of *M. synoviae* and other major *Mycoplasma* has not yet been systematically conducted and is the focus of this study.

PCR-based molecular detection is a common method for the rapid and accurate detection of pathogens (11). One established conventional PCR-based method for detecting *M. synoviae* is to detect the 16S rRNA gene or the sequences located in the intergenic spacer region (ISR) and the 23S rRNA gene (12–14). Other established conventional PCR-based methods used for detecting *M. synoviae* involve amplifying the conserved region of the *vlhA* gene. The amplified target products from *vlhA* vary in size. These *vlhA-*relevant detection methods are currently used more for strain typing (15, 16). In addition, Taqman quantitative real-time PCR (qPCR) analysis methods targeting 16S rRNA, ISR, or *vlhA*, and a SYBR green qPCR analysis method targeting 16S rRNA have been established (17–20). Subsequently, a SYBR green qPCR analysis method targeting *obg* and a combination of nested PCR and high-resolution melting curve analysis method targeting *obg* or *oppF* were established for differentiating MS-H from field strains (21–23). Furthermore, a mismatch amplification mutation assay targeting a single nucleotide polymorphism located in a HIT family protein coding gene has been developed for the differentiation of the MS1 vaccine strain from wild-type *M. synoviae* and MS-H vaccine strains (6). Thus, far, no qPCR detection method that targets *M. synoviae*-specific genes for clinical identification of *M. synoviae* has been established. To establish this method, the genes specific to *M. synoviae* relative to all publicly published biological species must be identified.

Only one SYBR green qPCR detection method for *M. synoviae* has been established, which has 16S rRNA as the diagnostic target (18). In view of the variability and/or possible poor specificity of a single gene, single target-based detection is sometimes not ideal for species identification. In the current study, the differences between different *M. synoviae* strains and between *M. synoviae* and other mycoplasma were analyzed using a variety of bioinformatics analyses. The specific *M. synoviae* genes, relative to the genes of all other published biological species, were first found. SYBR green qPCR detection methods targeting the specific genes were then established.

## Materials and Methods

### Ethics Statements

All of the animal experiments were performed in the Jiangsu Academy of Agricultural Sciences (JAAS) and were reviewed and approved by the Committee on the Ethics of Animal Experiments of JAAS. All of the experimental procedures conformed to the guidelines of Jiangsu Province Animal Regulations (Government Decree No. 45) in accordance with international law.

### Mycoplasma and Bacterial Culture Conditions

*M. synoviae, Mycoplasma gallisepticum, Mycoplasma gallinarum, Mycoplasma iowae*, and *Mycoplasma meleagridis* strains were cultured in modified Frey's medium at 37°C with 5% CO_2_ as described previously (24). *Staphylococcus aureus* strains were cultured in Todd-Hewitt medium at 37°C. Avian pathogenic *Escherichia coli* (APEC), *Salmonella enterica* subsp. *enterica* serovar Gallinarum biovar Pullorum (*S*. Pullorum), *Salmonella enterica* subsp. *enterica* serovar Gallinarum biovar Gallinarum (*S*. Gallinarum), and *Salmonella enterica* subsp. *enterica* serovar Typhimurium (*S*. Typhimurium) strains were cultured at 37°C in Luria-Bertani medium. The strains used in this study were passed fewer than five times *in vitro*.

### Whole Genome Comparisons

A total of 46 strains from 25 major animal *Mycoplasma* species/subspecies that had complete genome sequences and annotation information published in GenBank were selected and used for comparative genomic analysis. Among these 46 strains, there were 18 strains of nine avian *Mycoplasma* species, including six strains of *M. synoviae*. General characteristics of the complete genomes of the 46 strains of the 25 species/subspecies of *Mycoplasma* are listed in Supplementary Table 1.

The translated amino acid sequence sets corresponding to all protein-coding genes were obtained from the RefSeq annotation entries of the 46 strains in GenBank and used for core/pan gene analysis. The core/pan genes of the 46 *Mycoplasma* strains were clustered by CD-HIT (v 4.8.1) rapid clustering of similar proteins with a threshold of 50% pairwise identity and 70% length difference cut-off in amino acid sequence (25). The evolutionary relationships among the 46 strains of the 25 species/subspecies of *Mycoplasma* were characterized via analysis of 16S rRNA gene sequences and all single-copy core gene nucleotide sequences. All of the sequences mentioned above were extracted from the RefSeq of the 46 strains in GenBank. During similarity comparison and evolutionary tree analysis, multiple copies of 16S rRNA loci from one single mycoplasma strain were either retained with postfix, such as a, b, and c, to represent each copy or randomly selected and retained as only one copy. The 16S rRNA gene sequences and all single-copy core gene concatenated nucleotide sequences were aligned using MUSCLE integrated in MEGA X, respectively (26). The alignment results were used to construct phylogenetic trees using the maximum likelihood method based on the Tamura-Nei model in MEGA X with a bootstrap value of 1,000. Online software was used to calculate and draw a Venn diagram of the core-pan gene analysis results of six *M. synoviae* strains (http://bioinformatics.psb.ugent.be/webtools/Venn/). The homologies of complete genome sequences between every two *M. synoviae* strains were analyzed using BLASTN. The collinearity analysis and the number of SNPs (single nucleotide polymorphisms) and InDels (nucleotide insertion or deletion mutations) between two complete genome sequences of *M. synoviae* strains were analyzed using Mauve software (http://darlinglab.org/mauve/mauve.html). Insertion sequences (IS) were analyzed using the ISfinder tool (http://www-is.biotoul.fr).

### Identification of *M. synoviae*-Specific Functional Genes by Comparative Genomics

The functional genes shared by the six strains of *M. synoviae*, and specific to the remaining 40 strains of *Mycoplasma*, were obtained from the gene set obtained by the core/pan gene clustering in the previous step. A BLASTP similarity search (https://blast.ncbi.nlm.nih.gov/Blast.cgi) was subsequently performed on these gene encoded amino acid sequences using default parameters against the non-redundant protein sequences (nr) database to obtain specific functional genes with no homology in the database except for *M. synoviae*. The functional genes that were ultimately obtained were used as candidates for molecular diagnostic target genes. The candidate target genes were subjected to a BLASTN similarity search (https://blast.ncbi.nlm.nih.gov/Blast.cgi) against the standard database. Gene coverage > 40% and an E value < 10^−3^ were used as cutoffs to identify genes specific to *M. synoviae* and absent in all other species in the database at the nucleotide level (11).

### qPCR Detection Method Establishment

Primers targeting the genes of interest were designed using Primer3Plus (http://www.primer3plus.com/) and Primer Premier 5.00 (PREMIER Biosoft, http://www.premierbiosoft.com/). The specificity of the primers was determined using BLASTN against the standard databases. The qPCR assays were measured with the SYBR Premix Ex Taq II kit (TaKaRa, Japan) and run on the StepOnePlus^TM^ Real-Time PCR System (Applied Biosystems, US), according to the manufacturer's instructions.

Partial targeted gene sequences containing the qPCR primers were amplified by standard PCR using the primers designed as described above and cloned into the pMD-18T vector (TaKaRa, Japan) according to the user's manual. *E. coli* DH5α cells were used as the host for the recombinant plasmids. The copy number of recombinant plasmids was determined using an online calculator provided by the URI Genomics & Sequencing Center (http://cels.uri.edu/gsc/cndna.html) for determining the number of copies of a template. To evaluate sensitivity and reproducibility of the qPCR assays, the purified recombinant plasmids were diluted to 5 and 2.5 copies/reaction as well as continuously diluted by a factor of 10 to final concentrations of 10^10^ copies/reaction to 1 copy/reaction and used as the template to perform qPCR assays. Primers were also optimized by testing in the range of 100–500 nM.

To assess the specificity of the established qPCR method, the low pathogenic *M. gallinarum* (*n* = 1) and the major avian pathogenic bacteria/mycoplasmas, including *M. iowae* (*n* = 1), *M. meleagridis* (*n* = 1), *M. synoviae* (*n* = 20), *M. gallisepticum* (*n* = 3), *S. aureus* (*n* = 6), avian pathogenic *E. coli* (*n* = 4), *S*. Pullorum (*n* = 2), *S*. Gallinarum (*n* = 3), and *S*. Typhimurium (*n* = 3), were selected for analysis. The background information of these pathogens is provided in Supplementary Table 2. The last six pathogens mentioned above can cause arthritis in chickens. Clinically, it is often necessary to make a differential diagnosis of arthritis caused by these pathogens and *M. synoviae* (27–32). The genomic DNA of these 44 reference or clinical isolated strains of mycoplasma or bacteria was extracted. In addition, positive *M. synoviae*-infected chicken hock joint tissue samples, which were identified through culture-based pathogen isolation, genomic DNA extraction, and conventional PCR identification procedures reported in previous studies (33, 34), were selected for analysis. Chicken hock joint tissue samples positive for *M. synoviae* infection (*n* = 48) and negative control tissue samples (*n* = 10) were homogenized, and the mycoplasma genomic DNA was extracted to evaluate the specificity of the established qPCR method. Mycoplasma and bacterial genomic DNA was extracted using a Mycoplasma gDNA Mini Kit (BIOMIGA, China) and a MiniBEST Bacteria Genomic DNA Extraction Kit Ver.3.0 (TaKaRa, Japan), respectively, according to the manufacturers' instructions. All of the genomic and sample DNA concentrations were standardized to 1 ng/reaction. A sample was considered positive if the cycle threshold (C_T_) value was <36 and negative if the C_T_ value was undetectable. Samples with C_T_ values equal to or >36 were re-prepared and the detection concentration was increased by 10 times to re-test.

## Results

### Comparative Genomic Analysis of *M. synoviae* and Other *Mycoplasma* Species/Subspecies

The results of CD-HIT rapid clustering showed that there were 10,547 pan gene clusters among the 46 mycoplasma strains (Supplementary Table 3). After screening, a total of 16 protein-coding functional gene clusters (core gene clusters) were found to be shared by the 46 mycoplasma strains, of which three genes were multiple copies in some mycoplasmas and 13 genes were single copies in all 46 mycoplasmas (Table 1). Among these 16 genes, only *gap* was a double-copy in the six strains of *M. synoviae*, and the remaining 15 genes were single-copy in the six strains of *M. synoviae* (Table 1). In the pan gene clusters, 253 homologous gene clusters were shared by six *M. synoviae* strains and were absent in the other 40 mycoplasmas. Among these, 244 gene clusters were single-copy in all six strains of *M. synoviae*, and 9 gene clusters were multiple-copies in some of or all six *M. synoviae* strains (Supplementary Table 3).

**Table 1:** Core genes in 46 strains of 25 species/subspecies of *Mycoplasma*.

**Core genes which are not all single-copy in 46 strains**				
**Gene names**	**Descriptions**	**Locus tags in** ***M. synoviae*** **ATCC 25204 under Genbank accession number NZ_CP011096.1**	**% sequence similarities among 46 Mycoplasma strains**	**Copy numbers in each of 6** ***M. synoviae*** **strains**
*gap*	Type I glyceraldehyde-3-phosphate dehydrogenase	VY93_RS01150; VY93_RS01470	55.03–100.00	Two
*uvrA*	Excinuclease ABC subunit UvrA	VY93_RS00135	51.48–100.00	One
*rpsG*	30S ribosomal protein S7	VY93_RS00260	57.42–100.00	One
**Core genes which are all single-copy in 46 strains**				
**Gene names**	**Descriptions**	**Locus tags in** ***M. synoviae*** **ATCC 25204 under Genbank accession number NZ_CP011096.1**	**% sequence similarities among 46 Mycoplasma strains**	**Copy numbers in each of 6** ***M. synoviae*** **strains**
*lepA*	Elongation factor 4	VY93_RS02665	53.85–100.00	One
*atpD*	F0F1 ATP synthase subunit beta	VY93_RS02185	63.42–100.00	One
*eno*	Phosphopyruvate hydratase	VY93_RS00070	53.51–100.00	One
*tuf*	Elongation factor Tu	VY93_RS03630	71.83–100.00	One
*rplB*	50S ribosomal protein L2	VY93_RS03490	60.85–100.00	One
*rplP*	50S ribosomal protein L16	VY93_RS03470	55.07–100.00	One
*rpsM*	30S ribosomal protein S13	VY93_RS03170	54.55–100.00	One
*rplT*	50S ribosomal protein L20	VY93_RS03330	51.24–100.00	One
*rplN*	50S ribosomal protein L14	VY93_RS03455	55.74–100.00	One
*rpsS*	30S ribosomal protein S19	VY93_RS03485	60.92–100.00	One
*rpmI*	50S ribosomal protein L35	VY93_RS03325	50.79–100.00	One
type Z *rpsN*	Type Z 30S ribosomal protein S14	VY93_RS03440	57.38–100.00	One
*rpmJ*	50S ribosomal protein L36	VY93_RS03175	59.46–100.00	One

**Figure 1:**
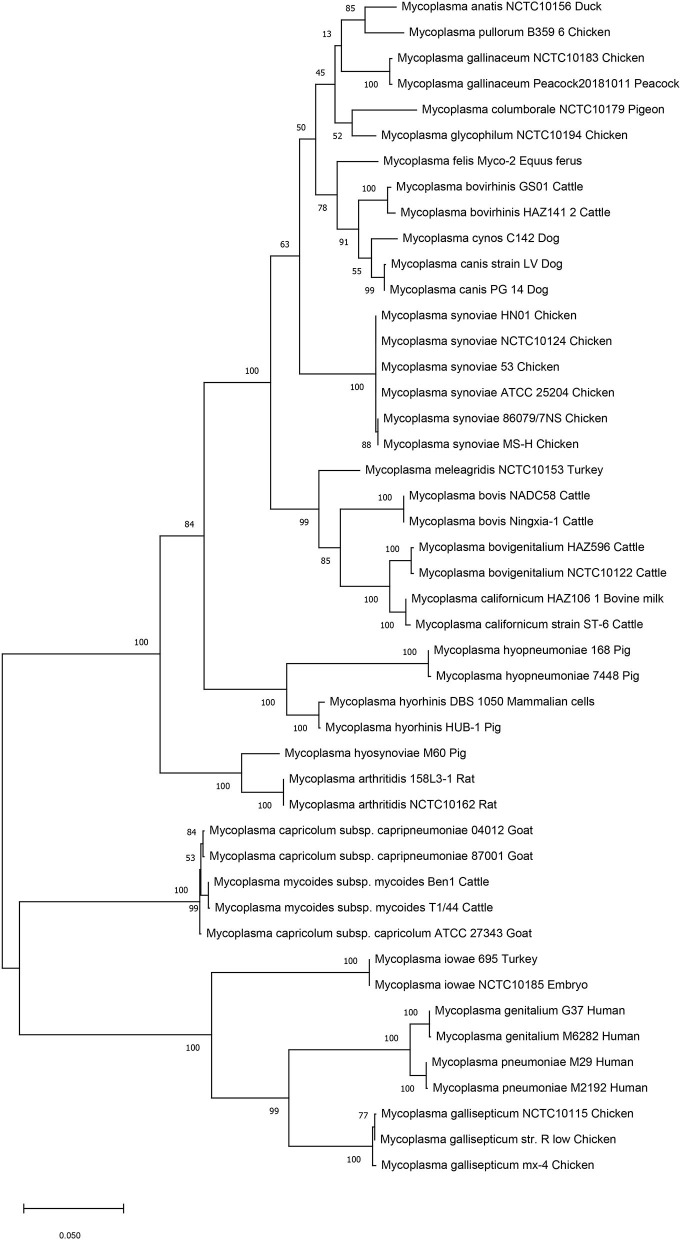
Phylogenetic tree based on the 16S rRNA gene. The DNA sequences of the 16S rRNA genes from 46 strains of 25 Mycoplasma species/subspecies were collected, aligned using MUSCLE, and analyzed via phylogenetic tree analysis using the maximum likelihood method. When more than one copy of 16S rRNA was in the genome of a mycoplasma strain, only one copy was randomly selected for analysis. Percent bootstrap values indicate support for internal nodes on the trees. The scale bar shows the distance.

**Figure 2:**
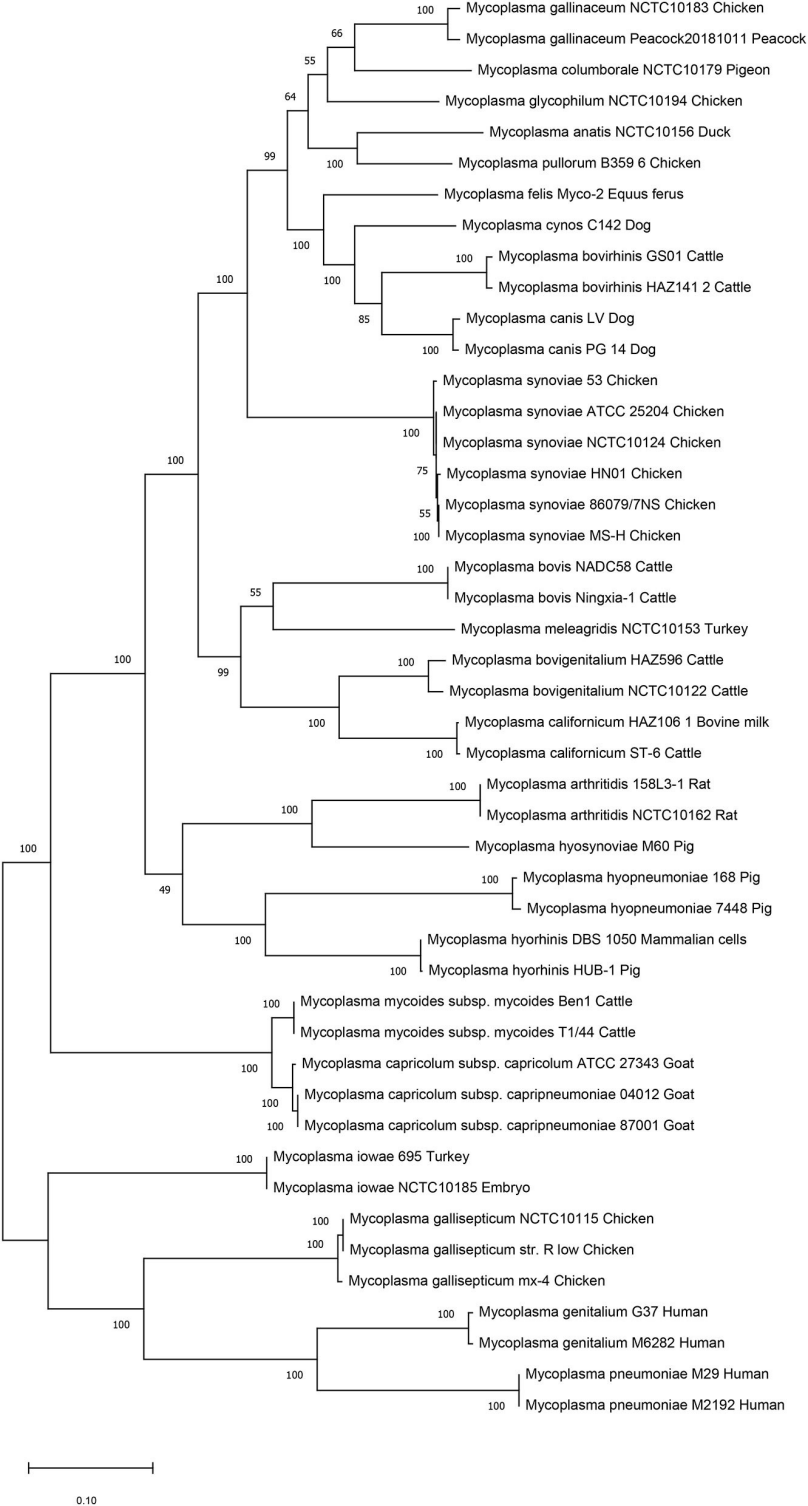
Phylogenetic tree based on 13 concatenated core-genes. The DNA sequences of 13 core-genes from 46 strains of 25 Mycoplasma species/subspecies were collected, concatenated in order, aligned using MUSCLE, and analyzed via phylogenetic tree analysis using the maximum likelihood method. Percent bootstrap values indicate support for internal nodes on the trees. The scale bar shows the distance.

We then performed genetic evolutionary analyses of *M. synoviae* relative to other major animal-derived mycoplasmas to study the specificity of *M. synoviae*. For homology comparison and evolutionary tree analysis, the nucleotide sequence of a gene is often more recognizable than the amino acid sequence it encodes. First, we used the DNA sequences of the 16S rRNA genes of each mycoplasma to perform homology comparisons and phylogenetic tree analysis. Given that most mycoplasmas have two or more copies of 16S rRNA, the phylogenetic tree based on the multi-copy 16S rRNA of all mycoplasma strains was not concise (Supplementary Figure 1). To facilitate the analysis, only one of the 16S rRNA multiple copies of each mycoplasma was randomly selected for phylogenetic tree analysis (Figure 1). The DNA sequences of 13 single-copy core genes from the 46 mycoplasma strains were subsequently selected for phylogenetic tree analysis (Figure 2). In general, the two phylogenetic trees were roughly the same.

Both phylogenetic tree analyses showed that *M. synoviae* was relatively distantly related to another major mycoplasma pathogen of chicken origin, *M. gallisepticum*. Phylogenetically, the two mycoplasmas were located in the two of the largest branches. The six strains of *M. synoviae* were placed into a relatively independent clade, and as a whole, were relatively closely related to a variety of mycoplasmas of avian origin, including *Mycoplasma gallinaceum, Mycoplasma columborale, Mycoplasma glycophilum, Mycoplasma anatis*, and *Mycoplasma pullorum*. Correspondingly, *M. gallisepticum* and *M. iowae* were closely related to two major human pathogenic mycoplasmas, *Mycoplasma genitalium* and *Mycoplasma pneumoniae*. In addition to *M. gallisepticum* and *M. iowae*, other avian mycoplasmas including *M. synoviae*, bovine mycoplasmas (except *Mycoplasma mycoides*), equine mycoplasma, and canine mycoplasmas were classified into one major clade.

The six strains of *M. synoviae* were divided into two and four category levels using 16S rRNA-based and 13 core gene-based phylogenetic tree analyses. The two evolutionary trees both showed that strains MS-H and 86079/7NS had higher homology between each other than the other four *M. synoviae* strains (Figures 1, 2). In the core gene-based phylogenetic tree analysis, the two subspecies of *Mycoplasma capricolum* were classified into one category, and then into the same category with *M. mycoides*. In the 16S rRNA-based phylogenetic tree analysis, the two subspecies of *M. capricolum* were not classified into the same lowest category. The three species of major swine pathogenic Mycoplasma were classified in the same category in the 13 core gene-based phylogenetic tree analyses, but not in the 16S rRNA-based analysis. In addition, *M. meleagridis* as well as canine mycoplasmas were classified into their own independent category in the 16S rRNA-based phylogenetic tree analysis, but not in the 13 core gene-based analysis.

In addition, the differences between *M. synoviae* and two species of pathogenic mycoplasmas that are characterized by causing mammalian arthritis and synovitis, *Mycoplasma arthritidis* and *Mycoplasma hyosynoviae*, were compared. Only two *M. arthritidis* strains (158L3-1 and NCTC10162) and one *M. hyosynoviae* strain (M60) had complete genome sequences and annotation information in GenBank. From the phylogenetic tree analysis, *M. synoviae* had low homology with *M. arthritidis* and *M. hyosynoviae*, while the homology relationship between *M. arthritidis* and *M. hyosynoviae* was very close. After screening the results of CD-HIT rapid clustering, there were 89 clusters in common between the six *M. synoviae* strains and two *M. arthritidis* strains, while none of the 89 clusters were absent in the rest of the 38 mycoplasma strains. There were 91 clusters in common between the six *M. synoviae* strains and one *M. hyosynoviae* strain, while none of the 91 clusters were absent in the rest of the 39 mycoplasma strains. There were 77 clusters in common among the six *M. synoviae* strains, two *M. arthritidis* strains, and one *M. hyosynoviae* strain, while none of the 77 clusters were absent in the rest of the 37 mycoplasma strains. Additionally, there was no cluster that was absent in MS-H but in common between five virulent *M. synoviae* strains and two *M. arthritidis* strains; between five virulent *M. synoviae* strains and one *M. hyosynoviae* strain; or among five *M. synoviae* strains, two *M. arthritidis* strains, and one *M. hyosynoviae* strain (Supplementary Table 3).

### Comparative Genomic Analysis of Six *M. synoviae* Strains

**Figure 3:**
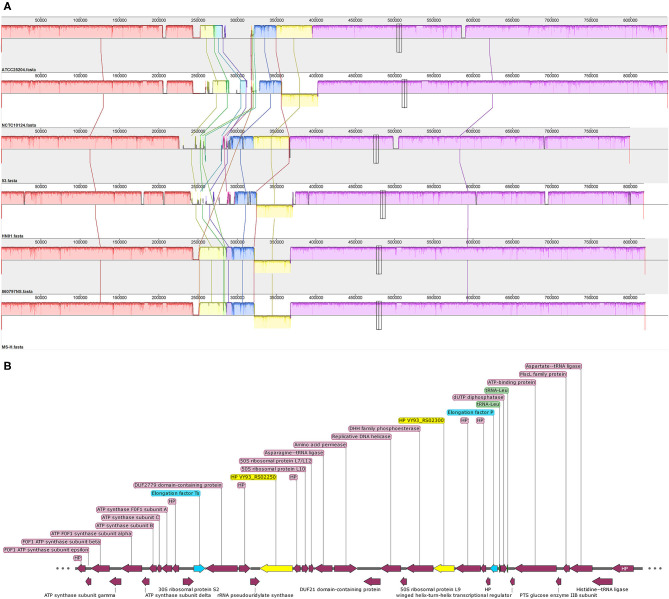
The collinearity analysis of six *M. synoviae* strains and the organization of two molecular diagnostic target genes and their upstream and downstream genes. **(A)** Alignment of the complete genome sequence of six *M. synoviae* strains. ATCC 25204 was used as the reference. Blocks of the same color connected by lines represent homologous regions. A block below the center line indicates an inversion region. The two merged thin and tall rectangles indicate the positions of VY93_R02250 and VY93_R02300 in each *M. synoviae*. **(B)** The organization of VY93_RS02250 and VY93_RS02300 and their upstream and downstream genes. Elongation factors Ts and P are marked in blue. VY93_RS02250 and VY93_RS02300 are marked in yellow. Two tRNA-Leu are marked in green. HP is the abbreviation of hypothetical protein.

Collinearity analysis showed that the genomes of the six *M. synoviae* strains had basic collinearity. In addition to the previously reported MS-H and 86079/7NS, which have a unique inversion of approximately 55 kb relative to ATCC 25204 (10, 35), the same inversion was also found in NCTC10124 and HN01 (Figure 3A). The two ends of the inversion were bound by the coding genes of asparagine ligase (VY93_RS01590) and tRNA-Gly (VY93_RS01775). There was a P80 family lipoprotein (VY93_01545 and VY93_RS01790) on the two wings of the inversion.

Pairwise complete genome nucleotide sequence coverage, identity, SNP number, and InDel number of ATCC 25204 or MS-H with the other five *M. synoviae* strains ranged between 96.00 and 100.00%, 99.09 and 99.98%, 279 and 6,216, and 19 and 472, respectively (Table 2 and Supplementary Table 4). In general, the differences between ATCC 25204 and NCTC10124 and between MS-H and 86079/7NS were much smaller than between the other two strains listed in Table 2.

**Table 2:** Overall comparisons of six *M. synoviae* strains.

**Strain names**	**Total length (bp)**	**Geographic origin**	**% of genome coverage (vs. ATCC 25204)**	**% of genome identity (vs. ATCC 25204)**	**% of genome coverage (vs. MS-H)**	**% of genome identity (vs. MS-H)**	**No. of SNPs (vs. ATCC 25204)**	**No. of InDels (vs. ATCC 25204)^**a**^**	**No. of SNPs (vs. MS-H)**	**No. of InDels (vs. MS-H)^**a**^**
ATCC 25204	846,495	USA	100.00	100.00	99.00	99.10	0	0 (0)	5979	472 (19)
NCTC10124	848,181	USA	100.00	99.98	99.00	99.10	279	53 (3)	5904	457 (17)
53	799,476	Brazil	96.00	99.14	96.00	99.22	6177	447 (10)	6216	426 (10)
HN01	817,087	China	98.00	99.09	98.00	99.19	5972	463 (19)	6059	469 (21)
86079/7NS	818,795	Australia	99.00	99.11	100.00	99.94	5849	462 (22)	399	19 (0)
MS-H	818,848	Australia	99.00	99.10	100.00	100.00	5979	472 (19)	0	0 (0)

a *The numbers in brackets show the numbers of InDels that more than 500 bp and do not located in the vlhA locus*.

**Figure 4:**
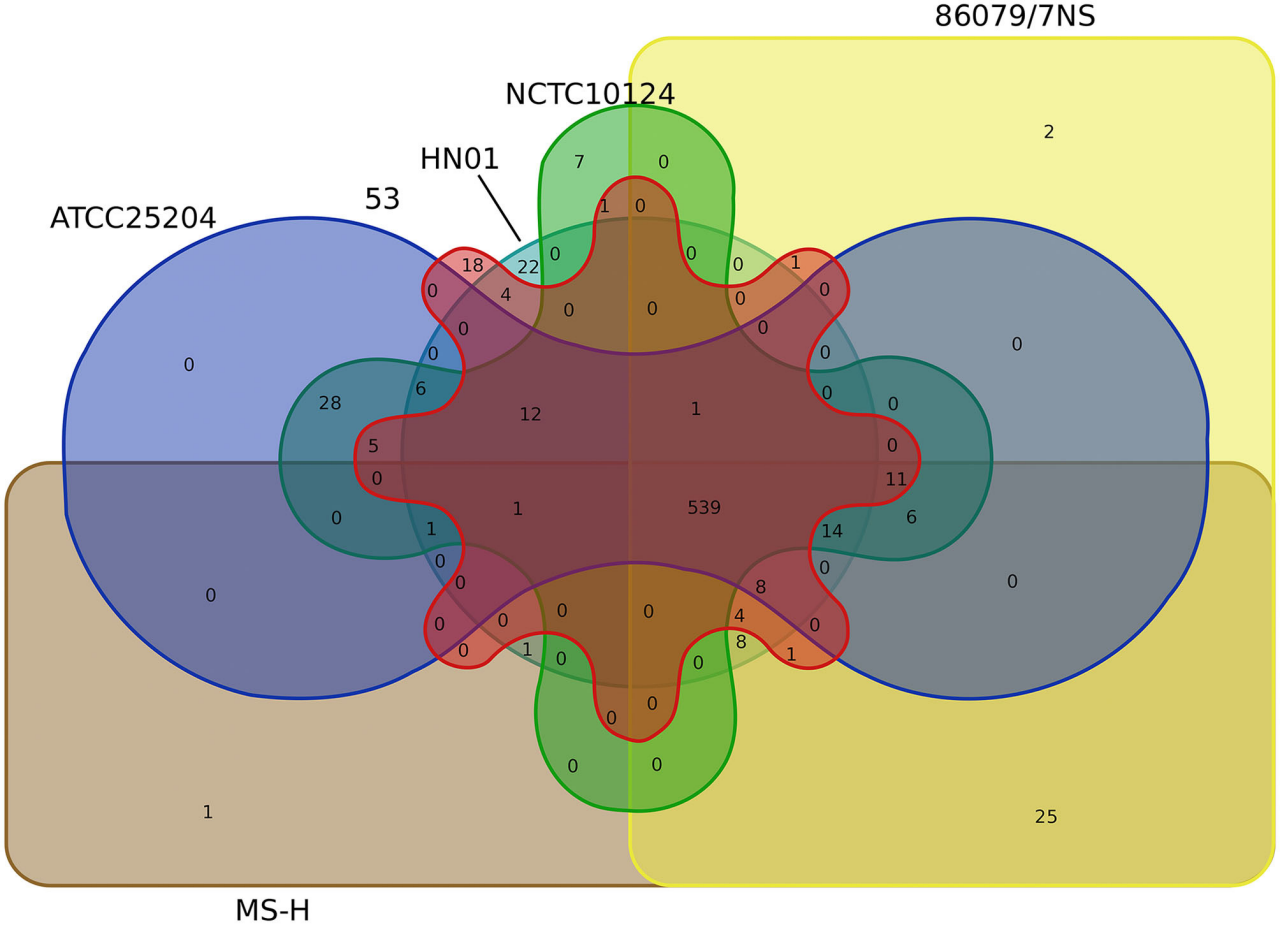
Venn diagram based on the core/pan gene cluster result of CD-HIT rapid clustering.

Core-pan gene analysis showed that there were 539 core clusters and 727 pan clusters in the six *M. synoviae* strains. Among them, only one cluster (cluster No. 10256, contained one gene, protein ID: WP_026365140.1, locus tag: MSH_RS02250, Supplementary Table 3) or two clusters that were unique to MS-H or 86079/7NS when compared to the other five strains, while 25 clusters were present in both MS-H and 86079/7NS but absent in the other four *M. synoviae* strains (Figure 4, Supplementary Table 5). These results indicate that MS-H and 86079/7NS have higher homology to each other relative to the other four strains. MSH_RS02250 encodes a hypothetical protein of 70 amino acids in MS-H (GenBank accession No. NZ_CP021129) and was subsequently subjected to a BLASTN similarity search. The result showed that the nucleotide sequence of MSH_RS02250 also exists in the corresponding position of the genomes of strains 53, ATCC 25204, 86079/7NS, and NCTC10124, whose GenBank accession Nos. are listed in Supplementary Table 1, but was not annotated as CDS in these strains. In addition, through SNP and InDel analysis, except in the *vlhA* locus, there were only point mutations and insertion/deletion mutations within 1 base in MS-H relative to its parent strain 86079/7NS (Supplementary Table 4). Therefore, MS-H had no specific functional genes, relative to the other five *M. synoviae* strains, that can be used for molecular diagnosis.

No cluster or seven clusters were unique to ATCC 25204 or NCTC10124 when compared to the other five strains, respectively, while 28 clusters were present in both ATCC 25204 and NCTC10124, but absent in the other four *M. synoviae* strains, indicating that ATCC 25204 and NCTC10124 have higher homology to each other relative to the other four strains. In contrast, strains 53 and HN01 had 18 and 22 unique clusters not found in the other five strains; while four clusters were present in both strains 53 and HN01 but absent in the other four *M. synoviae* strains, indicating that 53 and HN01 each have relatively distant homology with the other five strains (Figure 4).

A total of 253 clusters were found with common genes that were present in all six *M. synoviae* strains and absent in all of the other 40 mycoplasma strains, as mentioned above. Subsequently, 253 clusters containing genes were subjected to BLASTP similarity searches against the nr database in order to find unique functional genes in *M. synoviae* relative to species with known genome sequences. As a result, only six genes were found with amino acid sequences that could not be found by BLASTP in species other than *M. synoviae* (Table 3).

**Table 3:** *M. synoviae*-specific functional genes found by using BLASTP similarity search.

**Locus tags in ATCC 25204 under Genbank accession No. NZ_CP011096.1**	**Gene size (bp)**	**Descriptions**	**Sequence similarities among six** ***M. synoviae*** **(%)**					
			**ATCC 25204**	**NCTC10124**	**MS-H**	**86079/7NS**	**HN01**	**53**
VY93_RS00945	492	Hypothetical protein	100.00	100.00	95.71	95.71	97.55	97.55
VY93_RS00770	465	Hypothetical protein	100.00	100.00	97.97	97.97	97.97	98.65
VY93_RS01555	423	Hypothetical protein	96.43	96.43	100.00	100.00	93.38	96.43
VY93_RS02250	2,475	Hypothetical protein	100.00	100.00	99.88	99.88	99.39	99.51
VY93_RS02300	1,635	Hypothetical protein	100.00	100.00	99.43	99.43	98.67	97.91
VY93_RS02755	1,428	Hypothetical protein	99.37	99.37	99.37	99.37	98.74	100.00

### Construction of qPCR Detection Methods With *M. synoviae*-Specific Genes as Targets

Two of the six *M. synoviae*-specific genes with the largest gene sizes, VY93_RS02250 and VY93_RS02300, are located between two housekeeping genes, Elongation factor Ts and P. The upstream and downstream regions of these two genes mostly consist of functional genes related to protein synthesis and energy supply, and no IS element was found by gene function annotation in GenBank or analysis using the ISfinder (Figure 3B and Supplementary Table 6). The sequences of VY93_RS02250, VY93_RS02300, and their upstream and downstream genes in the six strains of *M. synoviae* were consistent, and the DNA sequence homology of each gene was not <98.17% as found by BLASTN analysis (Supplementary Table 6). There was only one exception: the 53 strain had a single-base frameshift mutation at the position corresponding to the open reading frame (ORF) of ATCC 25204 VY93_RS02305, which caused the position to be divided into two genes, MS53_RS02170 and MS53_RS02175 (Supplementary Tables 4, 6). The DNA sequence homology of the two strains in the ORF where VY_RS02305 is located is 99.02%. BLASTN analysis showed that VY93_RS02250 and VY93_RS02300 were specific to *M. synoviae*, the coverage of the nucleotide sequences of these two genes in different *M. synoviae* strains was 100%, and the identity was >99% (Supplementary Table 6). These two genes were selected as targets for molecular diagnosis in this study. VY93_RS02250 and VY93_RS02300 were used as targets for establishing subsequent qPCR detection methods.

The primer sequences for the two qPCR methods are listed in Table 4. The product sizes of the qPCR assay detecting VY93_RS02250 and VY93_RS02300 were 102 and 173 bp, respectively. The optimal concentrations of all primers were determined to be 200 nM (data not shown). The efficiency of the two qPCR assays was tested on control plasmids with different DNA copy numbers (10^10^-10 copies/reaction). Drawing a standard curve with the lg numbers of DNA copy number as the abscissa and C_T_ values as the ordinate, the correlation between DNA copy numbers and C_T_ values for VY93_RS02250 was *R*^2^ = 0.9997 (linear equation, y = −3.3393x + 38.267), and for VY93_RS02300 was *R*^2^ = 0.9989 (linear equation, y = −3.3269x + 38.074).

**Table 4:** Primers used for cloning and qPCR assays in this study.

**Target genes**	**Oligonucleotide sequences (5^′^ → 3^′^)^**a**^**	**Product sizes (bp)**
VY93_RS02250	F: ATTGCTTGTGCTAGCGTTTATCC	102
	R: ATTTGGTGGCGCTAAATTAACC	
VY93_RS02300	F: GCTGGCCCAAGATGTATTAGAC	173
	R: CCTTTTCATAGTTTGATTCAGGATT	

a *F, forward primer; R, reverse primer*.

**Figure 5:**
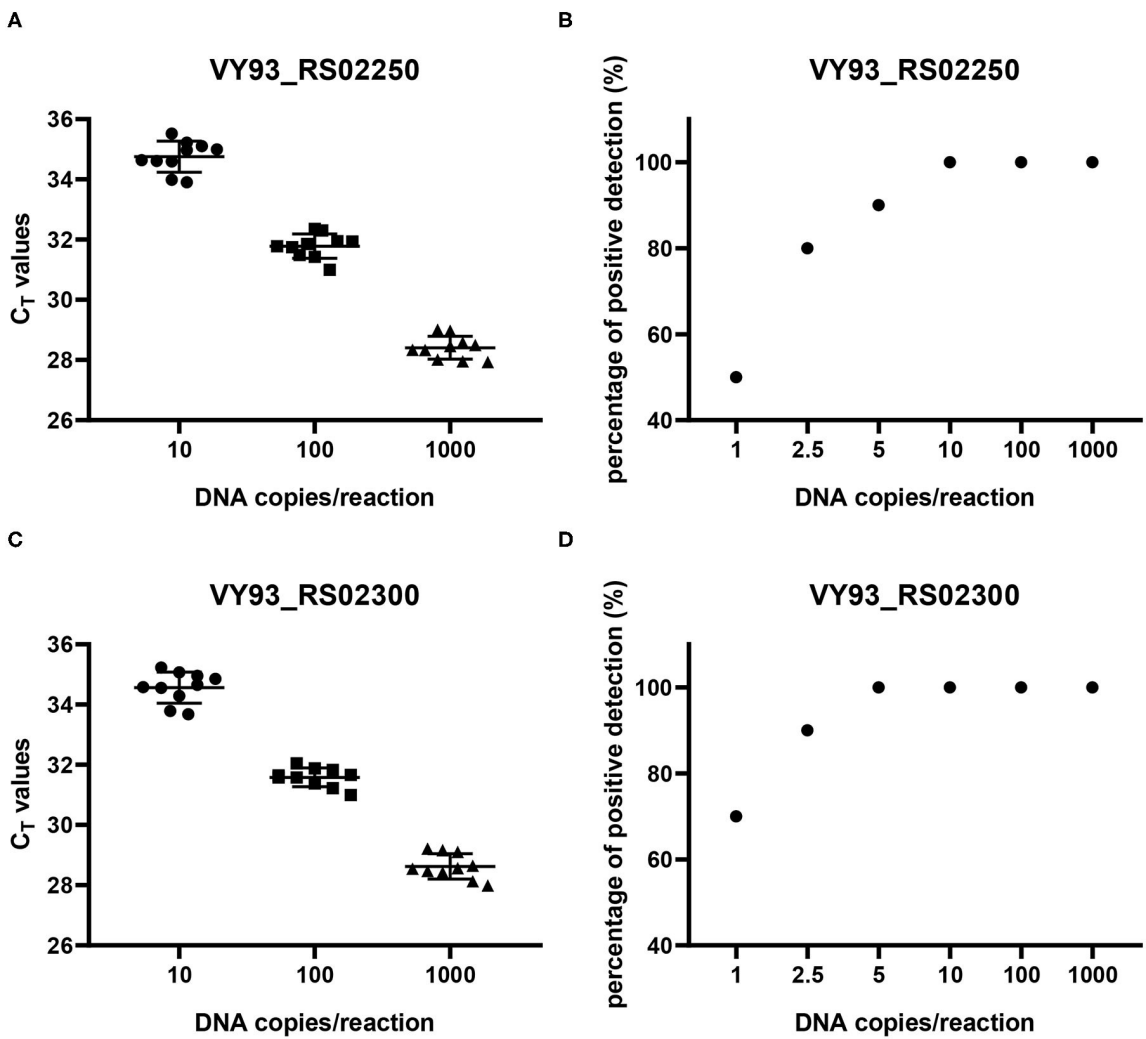
Reproducibility and sensitivity analyses of two qPCR methods. Reproducibility **(A,C)** was evaluated using corresponding control plasmids of three different concentrations of plasmid DNA with ten replicates in each concentration (10, 100, and 1,000 copies per reaction). Sensitivity **(B,D)** was evaluated using corresponding control plasmids of six different concentrations of plasmid DNA with 10 replicates in each concentration (1, 2.5, 5, 10, 100, and 1,000 copies per reaction).

The reproducibility of the two qPCR methods was evaluated with 10 replicates of each concentration of DNA copies in plasmids per reaction (Figures 5A,C). The results showed that when the number of DNA copies per reaction was 1,000, 100, and 10, the detected C_T_ values for genes VY93_RS02250 and VY93_RS02300 were 28.41 ± 0.38 and 28.62 ± 0.42, 31.78 ± 0.41 and 31.58 ± 0.32, and 34.75 ± 0.51 and 34.57 ± 0.52, respectively, which indicated good reproducibility of the two qPCR detection methods. Evaluation of the analytical sensitivity for the two targets showed 100% positive detection at a concentration of 10 DNA copies per reaction and variable detection percentage at a concentration of five or fewer DNA copies per reaction. Detection limits for VY93_RS02250 and VY93_RS02300 were 10 copies per reaction and 5 copies per reaction, respectively (Figures 5B,D).

The specificity test showed that when the genomic DNA of bacteria or mycoplasma strains were standardized to 1 ng/reaction, the positive amplified C_T_ values of the genes VY93_RS02250 and VY93_RS02300 of all 20 *M. synoviae* strains were 19.28 ± 0.95 and 19.60 ± 0.98, respectively; all other bacterial and mycoplasma strains had negative results (Supplementary Table 2). Clinical samples were also tested. When the primers targeting genes VY93_RS02250 and VY93_RS02300 were used for qPCR detection, and the extracted sample DNA standardized to 1 ng/reaction was used as the template, the test results showed that the C_T_ values of positive samples were ≤ 32.247, while the C_T_ value could not be detected in any of the negative samples (Supplementary Table 2).

## Discussion

Whole-genome sequencing and comparative genome analysis provides a valuable tool for better understanding *M. synoviae* genetics and for identifying unique genetic targets for assay development. In this study, we selected animal-derived *Mycoplasma* from public databases, particularly mycoplasma of avian origin. Core-pan gene cluster analysis showed that the selected 46 mycoplasma strains from 25 species/subspecies shared 13 single-copy common (core) genes. The differences between subspecies are generally smaller than the differences between species. The number of phylogenetic branches in multiple *M. synoviae* strains and the distinction between different *M. capricolum* subspecies and *M. mycoides* species indicate that the phylogenetic tree constructed with 13 core genes has higher resolution and more accuracy than the phylogenetic tree constructed with 16S rRNA alone at least within the same species when a large number of different species/subspecies of *Mycoplasma* are analyzed together. Concatenated MLST target genes from single bacteria species/subspecies or different species/subspecies of bacteria with close homology can also be used for phylogenetic tree analysis (36). However, in most cases, the target genes selected for construction of different bacterial/mycoplasma MLST typing methods are different (https://pubmlst.org/), which indicates that the concatenated MLST target genes are not suitable for phylogenetic tree analysis of multiple species/subspecies of bacteria/mycoplasma. Therefore, the core gene-based phylogenetic tree analysis method established in this study can be used in conjunction with the traditional 16S rRNA-based phylogenetic tree analysis to better analyze the evolutionary relationships among a wider range of different mycoplasma species/subspecies.

Until now, the complete genomes of six strains of *M. synoviae* from four continents have been published, which can provide a preliminary understanding of the similarities and differences of different *M. synoviae* strains from the same and different geographic locations. ATCC 25204 (GenBank accession No: NZ_CP011096.1) and NCTC10124 (GenBank accession No: NZ_LS991953.1) are from the same type strain WVU1583, which was isolated from a chicken joint in West Virginia, USA (6, 37). ATCC 25204 is stored in the American Type Culture Collection, while NCTC10124 is preserved in the Wellcome Trust Sanger Institute, United Kingdom. Whole-genome sequencing and annotation results showed that ATCC 25204 and NCTC10124 were comparable to MS-H and 86079/7NS in terms of genomic nucleotide sequence homology and the number of SNPs and InDels (Table 2). Furthermore, when comparing 86079/7NS with MS-H, 92.73% (*n* = 370/*n* = 399) of SNPs and 73.68% (*n* = 14/*n* = 19) of InDels were found to be located in the *vlhA* locus. When comparing ATCC 25204 with NCTC10124, 57.35% (*n* = 160/*n* = 279) of SNPs and 54.72% (*n* = 29/*n* = 53) of InDels were found to be located in the *vlhA* locus, which indicates that there are more mutations in functional genes between ATCC 25204 and NCTC10124 (Table 2 and Supplementary Table 4) other than virulence factor *vlhA*. This implies that there may be differences between ATCC 25204 and NCTC10124 in pathogenicity, growth, and other traits, indicating that variability is high in *M. synoviae*, and likely in all *Mycoplasma* strains, making the preservation of standard strains extremely challenging. Another major difference between ATCC 25204 and NCTC10124 was the unique 55 kb inversion relative to each other, which was first discovered in the same strain during the process of passage and preservation at different institutes. The specific molecular mechanism leading to this inversion is not yet known. Nevertheless, the inversion did not cause gene mutation, additions, or deletions. Thus, it may not affect the physiological and/or pathogenic functions of *M. synoviae*, nor does it affect the molecular diagnosis of *M. synoviae* gene sequences.

After core-pan gene analysis, only six *M. synoviae*-specific protein coding genes were found. However, none of these genes have been annotated with known functions. We also compared *M. synoviae* with *M. arthritidis* and *M. hyosynoviae* and did not find the common characteristic genes among these three species of mycoplasma. Mycoplasmas are bacteria with degenerative evolutionary characteristics (38, 39). Although *M. synoviae* has pathogenic properties that tend to cause infectious synovitis, it is assumed that the factors that affect these pathogenic properties may be due more to shared virulence factors or moonlighting proteins, such as VlhA as a cytoadhesin and enolase as a plasminogen/fibronectin binding protein (40, 41).

In *M. synoviae*, there is only one *vlhA* locus, which is a highly variable area and is flanked by identical homologs of type I glyceraldehyde-3-phosphate dehydrogenase (GAPDH) (10, 15, 42). This study found that, in addition to the two *M. synoviae* strains derived from the same parent strain, the number of SNPs obtained by comparing the six *M. synoviae* in pairs was about 6,000, and the number of InDels was about 450; however, the number of InDels that were larger than 500 bp and not located in the *vlhA* locus was only 10–22 (Table 2). MLST clustering and analysis, using the Maynard Smith method, suggests that there is minimal or no horizontal gene exchange among genomes from different *M. synoviae* strains (43). These provide favorable conditions for finding suitable target genes. Previous research has found that most of the loci containing strain-variable genes in the *M. synoviae* genome are associated with, and often flanked by, IS elements (35). Genes VY93_RS02250 and VY93_RS02300 are adjacent to a large number of single-copy housekeeping genes, such as two elongation factors and four ribosomal protein coding genes, and there are no IS elements around, indicating that losing the ORFs of these two genes would be very difficult. Our research also proved that 68 biological samples, including strains and positive disease materials, contained the DNA sequences of these two genes. Therefore, the two *M. synoviae*-specific functional genes are ideal molecular diagnostic targets.

It usually takes at least 1–3 weeks for *M. synoviae* to infect the host and stimulate the host to produce antibodies (18). Therefore, serological tests such as ELISA or hemagglutination inhibition (HI) cannot provide early diagnosis of infection caused by *M. synoviae*. The hemagglutination activity of *M. synoviae* is unstable (44). The *vlhA* encoding hemagglutinin itself is highly mutagenic and horizontally transfers between *M. synoviae* and other mycoplasmas, such as *M. gallisepticum* (45, 46). Therefore, the HI test both for antibody detection and pathogen detection is not suitable for clinical detection of *M. synoviae*. *M. synoviae* grows slowly. There is no selective medium for *M. synoviae* identification. Therefore, it is also difficult to rapidly diagnose *M. synoviae* through isolation and culture.

The use of multiple PCR methods to detect *M. synoviae* DNA in diseased tissues is an important method for rapid pathogen diagnosis. The established detection targets of conventional PCR or qPCR for *M. synoviae* detection are 16S rRNA, ISR, and *vlhA*, as described in the Introduction. Since isolating and culturing *M. synoviae* is relatively time-consuming, detecting *M. synoviae* pathogens directly from clinical tissue samples using highly sensitive qPCR has an advantage compared to conventional PCR. The primers that detect 16S rRNA of *M. gallisepticum* have been shown to be positive for *Mycoplasma imitans* (19, 47). Although primers for detecting *M. synoviae* 16S rRNA have not been found to detect other mycoplasmas non-specifically, 16S rRNA is indeed a gene that exists in all species, and there is a risk of non-specific detection. The same applies to detection primers that target ISR. The *vlhA* gene is highly mutated and horizontal transfer occurs among different mycoplasmas. For single-gene detection qPCR, SYBR green qPCR eliminates the need for probe synthesis compared to Taqman qPCR, so it is more economical. Previous studies have also reported that SYBR green qPCR is not inferior to Taqman qPCR in accuracy and sensitivity (48–50). In addition, only the SYBR green qPCR detection method for 16S rRNA has been established as of now. Many studies have shown that single target-based detection is sometimes not an ideal method for identifying bacterial or mycoplasma species (19, 36). Detection of multiple target genes can increase the sensitivity and specificity of strain identification, especially the identification of key strains in the research process. Molecular diagnostic methods for *M. synoviae*-specific genes have not yet been established. The two qPCR detection methods developed in this study provide more alternatives for molecular identification of *M. synoviae*.

In conclusion, in this study we established a phylogenetic tree based on the core genes of cross-mycoplasma species, which is a good supplement to the traditional 16S rRNA-based analysis. Through rapid CD-HIT clustering analysis, BLAST, collinearity analysis, and SNP and InDel analysis, we found a conserved DNA fragment in the *M. synoviae* genome, found two *M. synoviae*-specific functional genes in the DNA fragment, and established two SYBR green qPCR *M. synoviae* nucleic acid detection methods targeting these two genes, respectively, thus providing a new way for the rapid molecular identification of *M. synoviae*.

## Data Availability Statement

The original contributions presented in the study are included in the article/Supplementary Material, further inquiries can be directed to the corresponding author/s.

## Ethics Statement

The animal study was reviewed and approved by the Committee on the Ethics of Animal Experiments of the Jiangsu Academy of Agricultural Sciences (JAAS).

## Author Contributions

BX and XZ conceived and designed the experiments. BX, XC, FL, YS, HS, JZ, LS, and QP performed the experiments. BX and XC conducted bioinformatics analyses. BX, XC, and XZ analyzed the data. BX and XZ wrote the paper. CL offered suggestions. XZ revised the manuscript and coordinated the research. All authors contributed to the article and approved the submitted version.

## Conflict of Interest

The authors declare that the research was conducted in the absence of any commercial or financial relationships that could be construed as a potential conflict of interest.
