# Supplementary material for: Comparative Genomics of Mycoplasma synoviae and New Targets for Molecular Diagnostics
Source: Front Vet Sci. 2021 Feb 19;8:640067. doi: 10.3389/fvets.2021.640067 (PMC7933220; doi:10.3389/fvets.2021.640067)
Supplement: Supplementary file 1 [file Table_1.DOCX]

**SUPPLEMENTARY TABLE S1** General characteristics of the complete genomes of 46 strains of 25 species/subspecies of *Mycoplasma*

| Strains | Genbank accession No. | Total length (base pairs) | No. of genes | No. of CDSs^a^ | No. of CDSs with protein | No. of pseudogenes |
| --- | --- | --- | --- | --- | --- | --- |
| *Mycoplasma anatis* NCTC10156 | NZ_LR215035 | 978241 | 869 | 826 | 802 | 24 |
| *Mycoplasma arthritidis* 158L3-1 | NC_011025 | 820453 | 664 | 625 | 616 | 9 |
| *Mycoplasma arthritidis* NCTC10162 | NZ_LR215047 | 806209 | 643 | 604 | 596 | 8 |
| *Mycoplasma bovigenitalium* HAZ596 | NZ_AP017902 | 853553 | 742 | 702 | 690 | 12 |
| *Mycoplasma bovigenitalium* NCTC10122 | NZ_LR214970 | 841512 | 732 | 692 | 677 | 15 |
| *Mycoplasma bovirhinis* GS01 | NZ_CP024049 | 847985 | 713 | 671 | 656 | 15 |
| *Mycoplasma bovirhinis* HAZ141_2 | NZ_AP018135 | 948039 | 802 | 760 | 731 | 29 |
| *Mycoplasma bovis* NADC58 | NZ_CP022594 | 1098413 | 949 | 906 | 824 | 82 |
| *Mycoplasma bovis* Ningxia-1 | NZ_CP023663 | 1033629 | 877 | 834 | 750 | 84 |
| *Mycoplasma californicum* HAZ106_1 | NZ_AP018940 | 805917 | 673 | 633 | 627 | 6 |
| *Mycoplasma californicum* ST-6 | NZ_CP007521 | 793841 | 676 | 636 | 623 | 13 |
| *Mycoplasma canis* LV | NZ_CP011368 | 968791 | 758 | 716 | 687 | 29 |
| *Mycoplasma canis* PG 14 | NZ_CP014281 | 897443 | 701 | 659 | 627 | 32 |
| *Mycoplasma capricolum* subsp. *capricolum* ATCC 27343 | NC_007633 | 1010023 | 887 | 848 | 792 | 56 |
| *Mycoplasma capricolum* subsp. *capripneumoniae* 04012 | NZ_CP040917 | 1017343 | 902 | 863 | 699 | 164 |
| *Mycoplasma capricolum* subsp. *capripneumoniae* 87001 | NZ_CP006959 | 1017333 | 916 | 877 | 677 | 200 |
| *Mycoplasma columborale* NCTC10179 | NZ_LR215039 | 949548 | 796 | 756 | 747 | 9 |
| *Mycoplasma cynos* C142 | NC_019949 | 998123 | 806 | 766 | 733 | 33 |
| *Mycoplasma felis* Myco-2 | NZ_AP022325 | 841695 | 755 | 714 | 686 | 28 |
| *Mycoplasma gallinaceum* NCTC10183 | NZ_LR214950 | 1074838 | 832 | 790 | 771 | 19 |
| *Mycoplasma gallinaceum* Peacock20181011 | NZ_CP047225 | 1183913 | 916 | 872 | 832 | 40 |
| *Mycoplasma gallisepticum* mx-4 | NZ_CP044226 | 993340 | 811 | 769 | 718 | 51 |
| *Mycoplasma gallisepticum* NCTC10115 | NZ_LS991952 | 981408 | 803 | 762 | 704 | 58 |
| *Mycoplasma gallisepticum* str. R(low) | NC_004829 | 1012800 | 822 | 781 | 730 | 51 |
| *Mycoplasma genitalium* G37 | NC_000908 | 580076 | 564 | 522 | 512 | 10 |
| *Mycoplasma genitalium* M6282 | NC_018496 | 579504 | 570 | 528 | 454 | 74 |
| *Mycoplasma glycophilum* NCTC10194 | NZ_LR215024 | 973410 | 742 | 698 | 687 | 11 |
| *Mycoplasma hyopneumoniae* 168 | NC_017509 | 925576 | 747 | 711 | 680 | 31 |
| *Mycoplasma hyopneumoniae* 7448 | NC_007332 | 920079 | 741 | 705 | 667 | 38 |
| *Mycoplasma hyorhinis* DBS 1050 | NC_022807 | 837447 | 744 | 708 | 647 | 61 |
| *Mycoplasma hyorhinis* HUB-1 | NC_014448 | 839615 | 741 | 705 | 632 | 73 |
| *Mycoplasma hyosynoviae* M60 | NZ_CP008748 | 863547 | 677 | 638 | 626 | 12 |
| *Mycoplasma iowae* 695 | NZ_CP033512 | 1315476 | 1074 | 1039 | 1028 | 11 |
| *Mycoplasma iowae* NCTC10185 | NZ_LR215023 | 1278574 | 1034 | 999 | 990 | 9 |
| *Mycoplasma meleagridis* NCTC10153 | NZ_LR215042 | 644173 | 560 | 518 | 514 | 4 |
| *Mycoplasma mycoides* subsp. *mycoides* Ben1 | NZ_CP011260 | 1145921 | 1005 | 966 | 797 | 169 |
| *Mycoplasma mycoides* subsp. *mycoides* T1/44 | NZ_CP014346 | 1188848 | 1031 | 992 | 843 | 149 |
| *Mycoplasma pneumoniae* M29 | NZ_CP008895 | 857799 | 805 | 762 | 716 | 46 |
| *Mycoplasma pneumoniae* M2192 | NZ_CP010548 | 817169 | 767 | 725 | 687 | 38 |
| *Mycoplasma pullorum* B359_6 | NZ_CP017813 | 1007172 | 824 | 783 | 765 | 18 |
| *Mycoplasma synoviae* 53 | NC_007294 | 799476 | 719 | 675 | 632 | 43 |
| *Mycoplasma synoviae* 86079/7NS | NZ_CP029258 | 818795 | 725 | 682 | 647 | 35 |
| *Mycoplasma synoviae* ATCC 25204 | NZ_CP011096 | 846495 | 759 | 715 | 672 | 43 |
| *Mycoplasma synoviae* HN01 | NZ_CP034544 | 817087 | 740 | 696 | 658 | 38 |
| *Mycoplasma synoviae* MS-H | NZ_CP021129 | 818848 | 727 | 684 | 647 | 37 |
| *Mycoplasma synoviae* NCTC10124 | NZ_LS991953 | 848181 | 764 | 720 | 672 | 48 |

^a^ CDS, coding sequence.
