# Supplementary material for: Comparative Genomics of Mycoplasma synoviae and New Targets for Molecular Diagnostics
Source: Front Vet Sci. 2021 Feb 19;8:640067. doi: 10.3389/fvets.2021.640067 (PMC7933220; doi:10.3389/fvets.2021.640067)
Supplement: Supplementary file 5 [file Table_5.DOCX]

**SUPPLEMENTARY TABLE S5** A summary of cluster numbers included in different groups in Venn diagram

| Strains | Total numbers of elements | Lists of elements |
| --- | --- | --- |
| ATCC 25204  NCTC10124  53  HN01  86079/7NS  MS-H | 539 | Cluster 341, Cluster 1007, Cluster 719, Cluster 755, Cluster 381, Cluster 913, Cluster 844, Cluster 110, Cluster 866, Cluster 893, Cluster 997, Cluster 1050, Cluster 285, Cluster 823, Cluster 739, Cluster 1017, Cluster 978, Cluster 765, Cluster 1004, Cluster 40, Cluster 97, Cluster 825, Cluster 60, Cluster 253, Cluster 413, Cluster 784, Cluster 453, Cluster 858, Cluster 39, Cluster 877, Cluster 240, Cluster 1024, Cluster 31, Cluster 790, Cluster 36, Cluster 557, Cluster 21, Cluster 538, Cluster 53, Cluster 1044, Cluster 274, Cluster 999, Cluster 743, Cluster 742, Cluster 1014, Cluster 1065, Cluster 136, Cluster 813, Cluster 953, Cluster 104, Cluster 998, Cluster 252, Cluster 134, Cluster 412, Cluster 940, Cluster 172, Cluster 6, Cluster 12, Cluster 234, Cluster 129, Cluster 419, Cluster 130, Cluster 22, Cluster 203, Cluster 58, Cluster 728, Cluster 191, Cluster 198, Cluster 98, Cluster 15, Cluster 760, Cluster 287, Cluster 344, Cluster 255, Cluster 85, Cluster 342, Cluster 768, Cluster 1008, Cluster 128, Cluster 48, Cluster 845, Cluster 991, Cluster 147, Cluster 943, Cluster 220, Cluster 61, Cluster 183, Cluster 99, Cluster 74, Cluster 556, Cluster 245, Cluster 29, Cluster 1005, Cluster 979, Cluster 908, Cluster 586, Cluster 158, Cluster 842, Cluster 223, Cluster 91, Cluster 835, Cluster 938, Cluster 803, Cluster 230, Cluster 43, Cluster 914, Cluster 722, Cluster 974, Cluster 179, Cluster 379, Cluster 1025, Cluster 52, Cluster 476, Cluster 952, Cluster 20, Cluster 774, Cluster 758, Cluster 1045, Cluster 210, Cluster 1021, Cluster 923, Cluster 133, Cluster 122, Cluster 59, Cluster 502, Cluster 51, Cluster 281, Cluster 874, Cluster 264, Cluster 291, Cluster 1058, Cluster 137, Cluster 206, Cluster 859, Cluster 217, Cluster 1046, Cluster 226, Cluster 548, Cluster 1056, Cluster 400, Cluster 843, Cluster 1060, Cluster 166, Cluster 56, Cluster 464, Cluster 1016, Cluster 102, Cluster 8, Cluster 987, Cluster 737, Cluster 67, Cluster 832, Cluster 875, Cluster 504, Cluster 1009, Cluster 935, Cluster 814, Cluster 1019, Cluster 33, Cluster 283, Cluster 23, Cluster 86, Cluster 800, Cluster 236, Cluster 135, Cluster 72, Cluster 646, Cluster 42, Cluster 1000, Cluster 962, Cluster 741, Cluster 376, Cluster 1002, Cluster 681, Cluster 525, Cluster 335, Cluster 733, Cluster 638, Cluster 69, Cluster 167, Cluster 848, Cluster 127, Cluster 889, Cluster 614, Cluster 241, Cluster 915, Cluster 763, Cluster 753, Cluster 716, Cluster 38, Cluster 968, Cluster 732, Cluster 924, Cluster 106,Cluster 826, Cluster 410, Cluster 1020, Cluster 209, Cluster 424, Cluster 181, Cluster 782, Cluster 1037, Cluster 1027, Cluster 174, Cluster 1067, Cluster 853, Cluster 364, Cluster 164, Cluster 620, Cluster 756, Cluster 71, Cluster 933, Cluster 912, Cluster 193, Cluster 9, Cluster 77, Cluster 1047, Cluster 190, Cluster 993, Cluster 111, Cluster 44, Cluster 939, Cluster 822, Cluster 284, Cluster 1059, Cluster 1018, Cluster 96, Cluster 554, Cluster 156, Cluster 976, Cluster 408, Cluster 70, Cluster 885, Cluster 812, Cluster 1003, Cluster 180, Cluster 30, Cluster 225, Cluster 24, Cluster 801, Cluster 936, Cluster 906, Cluster 775, Cluster 1036, Cluster 1061, Cluster 68, Cluster 208, Cluster 796, Cluster 919, Cluster 854, Cluster 834, Cluster 333, Cluster 146, Cluster 182, Cluster 237, Cluster 789, Cluster 45, Cluster 18, Cluster 105, Cluster 222, Cluster 2, Cluster 888, Cluster 13, Cluster 969, Cluster 618, Cluster 90, Cluster 961, Cluster 187, Cluster 57, Cluster 989, Cluster 766, Cluster 54, Cluster 731, Cluster 849, Cluster 114, Cluster 547, Cluster 917, Cluster 787, Cluster 870, Cluster 895, Cluster 268, Cluster 503, Cluster 1034, Cluster 302, Cluster 779, Cluster 125, Cluster 747, Cluster 482, Cluster 310, Cluster 64, Cluster 1070, Cluster 94, Cluster 934, Cluster 154, Cluster 361, Cluster 904, Cluster 25, Cluster 1013, Cluster 830, Cluster 725, Cluster 734, Cluster 941, Cluster 927, Cluster 80, Cluster 838, Cluster 248, Cluster 258, Cluster 770, Cluster 415, Cluster 442, Cluster 489, Cluster 299, Cluster 517, Cluster 304, Cluster 820, Cluster 228, Cluster 195, Cluster 1069, Cluster 84, Cluster 817, Cluster 963, Cluster 119, Cluster 929, Cluster 905, Cluster 214, Cluster 664, Cluster 11, Cluster 804, Cluster 1040, Cluster 886, Cluster 970, Cluster 10, Cluster 282, Cluster 1048, Cluster 759, Cluster 578, Cluster 654, Cluster 116, Cluster 746, Cluster 265, Cluster 169, Cluster 257, Cluster 188, Cluster 881, Cluster 534, Cluster 982, Cluster 1028, Cluster 762, Cluster 46, Cluster 996, Cluster 441, Cluster 916, Cluster 239, Cluster 32, Cluster 1033, Cluster 337, Cluster 7, Cluster 903, Cluster 150, Cluster 250, Cluster 66, Cluster 75, Cluster 1057, Cluster 428, Cluster 126, Cluster 928, Cluster 83, Cluster 269, Cluster 807, Cluster 839, Cluster 931, Cluster 740, Cluster 1054, Cluster 869, Cluster 395, Cluster 155, Cluster 65, Cluster 711, Cluster 828, Cluster 358, Cluster 103, Cluster 527, Cluster 1041, Cluster 1077, Cluster 780, Cluster 3, Cluster 202, Cluster 120, Cluster 793, Cluster 495, Cluster 1001, Cluster 224, Cluster 894, Cluster 229, Cluster 211, Cluster 715, Cluster 16, Cluster 249, Cluster 861, Cluster 26, Cluster 1049, Cluster 55, Cluster 841, Cluster 367, Cluster 995, Cluster 47, Cluster 810, Cluster 95, Cluster 781, Cluster 1029, Cluster 78, Cluster 19, Cluster 788, Cluster 14, Cluster 251, Cluster 199, Cluster 767, Cluster 806, Cluster 92, Cluster 288, Cluster 836, Cluster 972, Cluster 37, Cluster 752, Cluster 1064, Cluster 244, Cluster 891, Cluster 123, Cluster 145, Cluster 429, Cluster 189, Cluster 583, Cluster 82, Cluster 911, Cluster 1031, Cluster 117, Cluster 898, Cluster 196, Cluster 948, Cluster 336, Cluster 785, Cluster 184, Cluster 798, Cluster 34, Cluster 431, Cluster 4, Cluster 62, Cluster 112, Cluster 178, Cluster 899, Cluster 27, Cluster 868, Cluster 851, Cluster 185, Cluster 1010, Cluster 902, Cluster 331, Cluster 163, Cluster 932, Cluster 818, Cluster 216, Cluster 143, Cluster 773, Cluster 918, Cluster 507, Cluster 138, Cluster 50, Cluster 777, Cluster 1032, Cluster 951, Cluster 791, Cluster 559, Cluster 624, Cluster 689, Cluster 644, Cluster 139, Cluster 819, Cluster 964, Cluster 896, Cluster 124, Cluster 360, Cluster 152, Cluster 1006, Cluster 262, Cluster 243, Cluster 1043, Cluster 778, Cluster 867, Cluster 93, Cluster 235, Cluster 797, Cluster 87, Cluster 745, Cluster 957, Cluster 771, Cluster 942, Cluster 1023, Cluster 1011, Cluster 860, Cluster 786, Cluster 162, Cluster 115, Cluster 17, Cluster 131, Cluster 149, Cluster 678, Cluster 314, Cluster 549, Cluster 837, Cluster 218, Cluster 35, Cluster 890, Cluster 901, Cluster 101, Cluster 593, Cluster 118, Cluster 28, Cluster 197, Cluster 876, Cluster 920, Cluster 921, Cluster 5, Cluster 414, Cluster 160, Cluster 194, Cluster 930, Cluster 142, Cluster 983, Cluster 1015, Cluster 857, Cluster 49, Cluster 950, Cluster 168, Cluster 850, Cluster 349, Cluster 792, Cluster 88, Cluster 63, Cluster 714 |
| ATCC25204  53  HN01  86079/7NS  MS-H | 8 | Cluster 659, Cluster 706, Cluster 1480, Cluster 81, Cluster 256, Cluster 73, Cluster 1124, Cluster 490 |
| ATCC25204  NCTC10124  53  HN01  86079/7NS | 1 | Cluster 1114 |
| ATCC25204  NCTC10124  53  86079/7NS  MS-H | 11 | Cluster 1652, Cluster 355, Cluster 1555, Cluster 312, Cluster 1276, Cluster 1623, Cluster 417, Cluster 1081, Cluster 1096, Cluster 1669, Cluster 438 |
| ATCC25204  NCTC10124  53  HN01  MS-H | 1 | Cluster 1638 |
| ATCC 25204  NCTC10124  HN01  86079/7NS  MS-H | 14 | Cluster 596, Cluster 546, Cluster 1672, Cluster 404, Cluster 518, Cluster 1109, Cluster 232, Cluster 1148, Cluster 1089, Cluster 175, Cluster 1080, Cluster 76, Cluster 505, Cluster 1112 |
| 53  HN01  86079/7NS  MS-H | 4 | Cluster 2229, Cluster 1698, Cluster 480, Cluster 2106 |
| ATCC 25204  NCTC10124  53  HN01 | 12 | Cluster 1786, Cluster 370, Cluster 1429, Cluster 2305, Cluster 1681, Cluster 1948, Cluster 2068, Cluster 2314, Cluster 1022, Cluster 108, Cluster 1827, Cluster 2224 |
| ATCC 25204  NCTC10124  86079/7NS  MS-H | 6 | Cluster 1711, Cluster 522, Cluster 907, Cluster 685, Cluster 2302, Cluster 582 |
| ATCC 25204  NCTC10124  HN01  MS-H | 1 | Cluster 1842 |
| 53  86079/7NS  MS-H | 1 | Cluster 3366 |
| ATCC 25204  NCTC10124  53 | 5 | Cluster 2739, Cluster 3329, Cluster 3163, Cluster 3339, Cluster 3325 |
| HN01  86079/7NS  MS-H | 8 | Cluster 1461, Cluster 2838, Cluster 3102, Cluster 3246, Cluster 3041, Cluster 3283, Cluster 3162, Cluster 1668 |
| ATCC 25204  NCTC10124  HN01 | 6 | Cluster 3363, Cluster 2899, Cluster 485, Cluster 1062, Cluster 3335, Cluster 1577 |
| 53  86079/7NS | 1 | Cluster 276 |
| 53  HN01 | 4 | Cluster 1637, Cluster 1426, Cluster 4367, Cluster 6988 |
| NCTC10124  53 | 1 | Cluster 6683 |
| 86079/7NS  MS-H | 25 | Cluster 6296, Cluster 3449, Cluster 3304, Cluster 6830, Cluster 6420, Cluster 6154, Cluster 1549, Cluster 4199, Cluster 6760, Cluster 1498, Cluster 456, Cluster 6234, Cluster 6225, Cluster 5299, Cluster 6872, Cluster 6546, Cluster 6419, Cluster 4458, Cluster 6636, Cluster 6578, Cluster 6903, Cluster 5937, Cluster 6383, Cluster 6961, Cluster 2379 |
| ATCC 25204  NCTC10124 | 28 | Cluster 5794, Cluster 6917, Cluster 6859, Cluster 4895, Cluster 5152, Cluster 6601, Cluster 3701, Cluster 670, Cluster 4601, Cluster 4281, Cluster 6966, Cluster 6927, Cluster 6860, Cluster 6904, Cluster 5779, Cluster 5343, Cluster 3731, Cluster 6885, Cluster 6955, Cluster 5816, Cluster 6274, Cluster 3367, Cluster 6817, Cluster 6979, Cluster 6738, Cluster 6235, Cluster 5416, Cluster 6957 |
| HN01  MS-H | 1 | Cluster 4868 |
| 53 | 18 | Cluster 8551, Cluster 6107, Cluster 10110, Cluster 6126, Cluster 7029, Cluster 7096, Cluster 10048, Cluster 9928, Cluster 8308, Cluster 9718, Cluster 7583, Cluster 10305, Cluster 10109, Cluster 9844, Cluster 9558, Cluster 6871, Cluster 1803, Cluster 10156 |
| 86079/7NS | 2 | Cluster 8960, Cluster 10082 |
| HN01 | 22 | Cluster 9121, Cluster 9204, Cluster 9954, Cluster 9274, Cluster 9974, Cluster 10339, Cluster 9961, Cluster 9055, Cluster 9333, Cluster 8107, Cluster 9929, Cluster 6098, Cluster 10355, Cluster 9719, Cluster 10492, Cluster 8482, Cluster 9428, Cluster 10329, Cluster 132, Cluster 7932, Cluster 10286, Cluster 9815 |
| MS-H | 1 | Cluster 10262 |
| NCTC10124 | 7 | Cluster 1074, Cluster 393, Cluster 2323, Cluster 1026, Cluster 645, Cluster 1526, Cluster 1053 |
