# Supplementary figures and images for: Comparative Genomics of Mycoplasma synoviae and New Targets for Molecular Diagnostics
Source: Front Vet Sci. 2021 Feb 19;8:640067. doi: 10.3389/fvets.2021.640067 (PMC7933220; doi:10.3389/fvets.2021.640067)

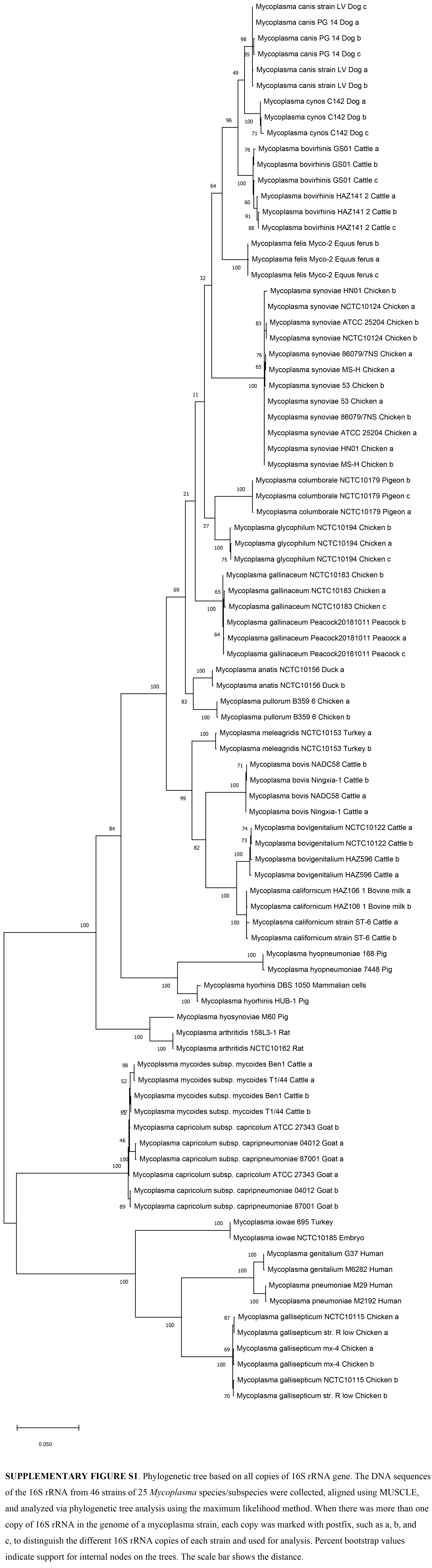

Supplement: Supplementary file 7 [file Image_1.TIF]
